# Ataxin-3 like (ATXN3L), a member of the Josephin family of deubiquitinating enzymes, promotes breast cancer proliferation by deubiquitinating Krüppel-like factor 5 (KLF5)

**DOI:** 10.18632/oncotarget.4128

**Published:** 2015-05-12

**Authors:** Fei Ge, Wenlin Chen, Junying Qin, Zhongmei Zhou, Rong Liu, Linlin Liu, Jing Tan, Tianning Zou, Hongyuan Li, Guosheng Ren, Ceshi Chen

**Affiliations:** ^1^ Key Laboratory of Animal Models and Human Disease Mechanisms of the Chinese Academy of Sciences and Yunnan Province, Kunming Institute of Zoology, Chinese Academy of Sciences, Kunming, Yunnan, China; ^2^ Chongqing Key Laboratory of Molecular Oncology and Epigenetics, The First Affiliated Hospital of Chongqing Medical University, Chongqing, China; ^3^ Department of Breast Surgery, The 3rd Affiliated Hospital of Kunming Medical University, Kunming, Yunnan, China; ^4^ Laboratory for Conservation and Utilization of Bioresource, Yunnan University, Kunming, China

**Keywords:** ATXN3L, KLF5, DUB, breast cancer

## Abstract

The Krüppel-like factor 5 (KLF5) has been suggested to promote breast cell proliferation, survival and tumorigenesis. KLF5 protein degradation is increased by several E3 ubiquitin ligases, including WWP1 and SCF^Fbw7^, through the ubiquitin-proteasome pathway. However, the deubiquitinase (DUB) of KLF5 has not been demonstrated. In this study, we identified ATXN3L as a KLF5 DUB by genome-wide siRNA screening. ATXN3L directly binds to KLF5, decreasing its ubiquitination and thus degradation. Functionally, knockdown of ATXN3L inhibits breast cancer cell proliferation partially through KLF5. These findings reveal a previously unrecognized role of ATXN3L in the regulation of KLF5 stability in breast cancer. ATXN3L might be a therapeutic target for breast cancer.

## INTRODUCTION

Krüppel-like factor 5 (KLF5) is a zinc finger transcription factor regulating gene transcription, cell proliferation [1], cell cycle [2], apoptosis [3], cell migration [4] and stem cell renewal [5]. Previous studies showed that KLF5 is highly expressed in estrogen receptor (ER)α-negative basal-type breast cancer [6]. High *KLF5* mRNA and protein levels predict unfavorable clinical outcomes for breast cancer patients [7]. Our previous studies demonstrated that KLF5 promoted cell survival, proliferation and tumor growth partially through direct up-regulation of fibroblast growth factor binding protein 1(*FGF-BP*) [1] and microsomal prostaglandin E2 synthase 1 (*mPGES1*) [8] gene transcription. These findings define KLF5 as an oncogenic transcription factor and a potential therapeutic target for basal-type breast cancer.

Ubiquitination is a reversible post-translational modification of proteins. KLF5 is an unstable protein with a short half-life. It can be degraded through the ubiquitin-dependent proteasome pathway [9] and is ubiquitinated by the E3 ligase activity of WWP1 [10], SCF^Fbw7^ [11], Smurf2 [12] and EFP [13]. Consistently, these E3 ligases inhibit the expression of KLF5 and cell growth in breast cancer.

Deubiquitinating enzymes (DUBs)are emerging as important regulators of cancer [14, 15]. DUBs are a family of approximately 98 enzymes that catalyze the removal of ubiquitin from protein substrates and regulate protein functions [16]. DUBs can be grouped into 6 subfamilies: Ubiquitin-Specific Proteases (USPs), Ubiquitin Carboxy-terminal Hydrolases (UCHs), Ovarian-Tumor Proteases (OTUs), Machado-Joseph Disease Protein Domain Proteases, JAMM/MPN domain-associated Metallopeptidases (JAMMs) and Monocyte Chemotactic Protein-Induced Protein (MCPIP) family [14]. The DUBs for KLF5 have not been reported so far.

Since KLF5 DUBs may stabilize the KLF5 protein and promote breast cancer, we screened a siRNA library including 87 human DUBs. We identified ATXN3L as a candidate KLF5 DUB because knockdown of ATXN3L decreased KLF5 protein levels and inhibited breast cancer cell proliferation.

## RESULTS

### Identification of ATXN3L as a candidate DUB for KLF5

**Figure 1:**
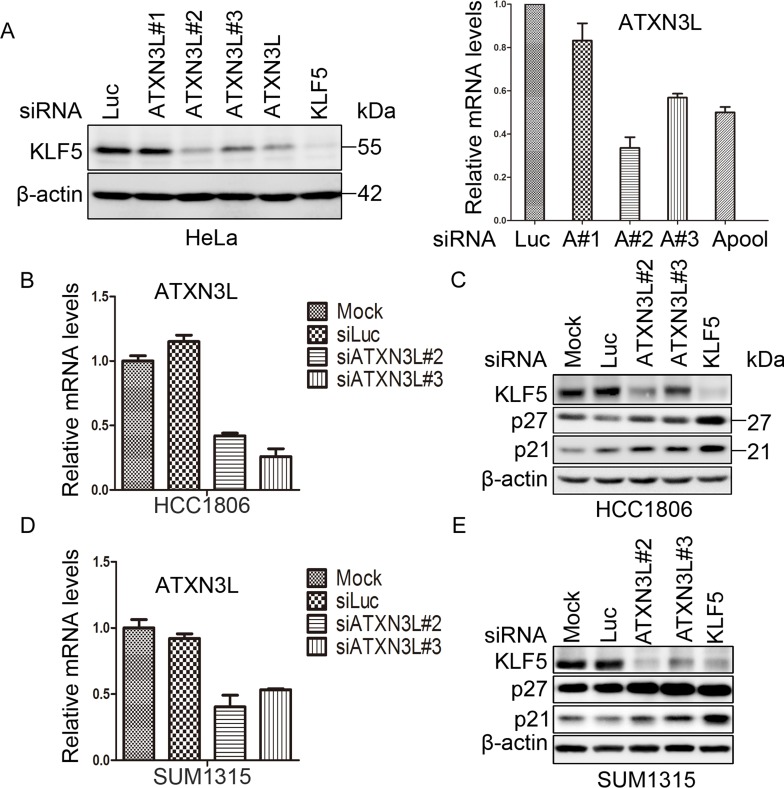
ATXN3L is a candidate DUB for KLF5 **A.** Validation of ATXN3L as a candidate KLF5 DUB in HeLa cells. ATXN3L was knocked down using three individual (#1 did not work well) or pooled siRNAs. The knockdown efficiency, as measured by RT-qPCR, is shown on the right. Luciferase (Luc) siRNA was used as the negative control. The KLF5 protein was detected by WB. β-actin served as the loading control. **B.** The knockdown effect of ATXN3L using two independent siRNAs in HCC1806 cells was determined by RT-qPCR at the mRNA level. **C.** Knockdown of ATXN3L using two independent siRNAs in HCC1806 cells decreased the KLF5 protein levels and increased the p27 and p21 protein levels. KLF5 siRNA was used as the positive control. **D.** The knockdown effect of ATXN3L using two independent siRNAs in SUM1315 cells was determined by RT-qPCR at the mRNA level. **E.** Knockdown of ATXN3L using two independent siRNAs in SUM1315 cells decreased the KLF5 protein levels and increased the p27 and p21 protein levels. KLF5 siRNA was used as the positive control.

In order to identify KLF5 DUBs, we screened a siRNA library consisting of siRNA pools (a mixture of three siRNAs per gene) against 87 human DUBs. We knocked down individual human DUBs in HeLa cells for two days and detected the KLF5 protein levels by WB. ATXN3L was identified as a candidate KLF5 DUB because the siRNA pool, and 2 out of 3 individual siRNA, against ATXN3L dramatically decreased the KLF5 protein level in HeLa (Figure 1A). ATXN3L is a novel DUB whose function and mechanism have not been well characterized.

To further confirm whether ATXN3L knockdown decreases KLF5 protein stability in human breast cancer cells, we knocked down ATXN3L in HCC1806 and SUM1315 (two basal type triple negative breast cancer cell lines), in which KLF5 is highly expressed (Figure 1C & 1E). We used quantitative RT-PCR to validate the knockdown efficiency of ATXN3L siRNA (#2 and #3) since several anti-ATXN3L antibodies failed to detect the endogenous ATXN3L protein (data not shown). Each ATXN3L siRNAs reduced the *ATXN3L* mRNA expression levels for at least 50% compared to the negative control in both HCC1806 and SUM1315 cells (Figure 1B & 1D). As expected, knockdown of ATXN3L dramatically decreased the endogenous KLF5 protein levels in both cell lines (Figure 1C & 1E).

KLF5 has previously been shown to inhibit the expression of the cell cycle-dependent kinase inhibitors p21 [17] and p27 [2]. Confirming these findings, knockdown of KLF5 upregulated p27 and p21 protein levels in two cancer cell lines as shown in Figure 1C & 1E. Furthermore, knockdown of ATXN3L by two different siRNAs also upregulated p27 and p21 protein levels in these cell lines (Figure 1C & 1E).

### ATXN3L interacts with KLF5

**Figure 2:**
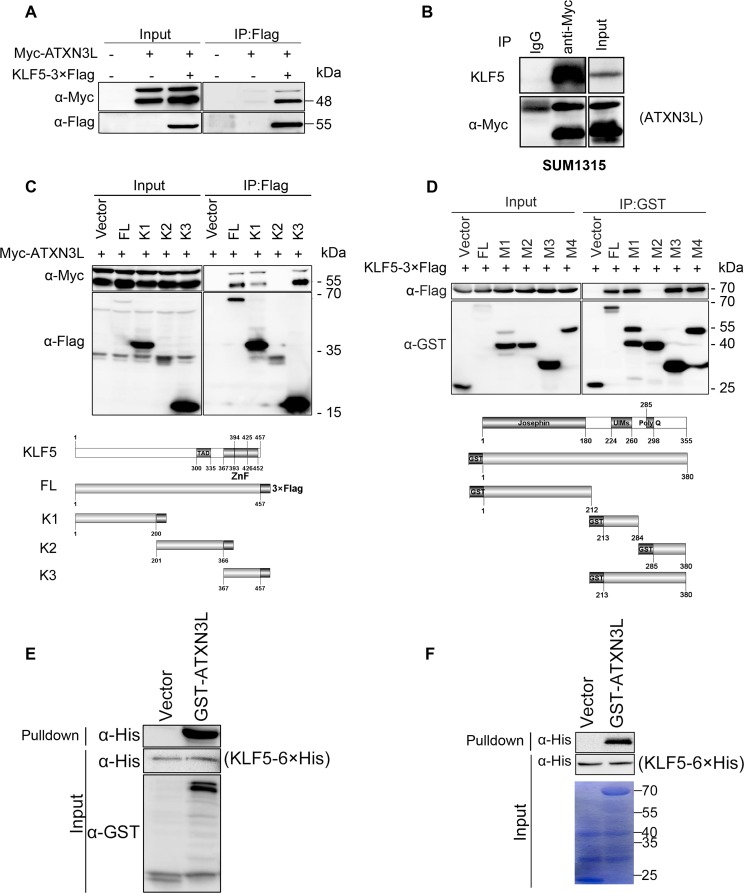
Protein interaction between ATXN3L and KLF5 **A.** Myc-ATXN3L and KLF5-3×Flag were specifically co-immunoprecipitated. Myc-ATXN3L and KLF5-3×Flag were expressed in HEK293FT cells. When KLF5-3×Flag was immunoprecipitated by the Flag-M2 beads, Myc-ATXN3L was specifically co-immunoprecipitated. Myc-ATXN3L showed two bands by WB. **B.** Myc-ATXN3L was transiently overexpressed in SUM1315 cells, Myc-ATXN3L was specifically co-immunoprecipitated by the anti-Myc antibody. Endogenous KLF5 proteins were specifically co-immunoprecipitated. **C.** Both N-terminal and C-terminal KLF5 protein fragments interacted with ATXN3L. Myc-ATXN3L and C-terminal 3×Flag-tagged full-length KLF5 or its mutants (a schematic diagram is shown below the panel) were expressed in HEK293FT cells. Immunoprecipitation was performed with Flag-M2 beads and the Myc-ATXN3L protein was detected using the anti-Myc antibody. **D.** Both N-terminal and C-terminal ATXN3L protein fragments interacted with KLF5. KLF5-3×Flag and GST fused full-length ATXN3L or its deletion mutants (a schematic diagram is shown below the panel) were expressed in HEK293FT cells. GST fusion proteins were precipitated with glutathione beads and the KLF5-3×Flag protein was detected using the anti-Flag antibody. **E.** Mammalian GST-ATXN3L directly interacted with KLF5-6×His as measured by the GST pull-down assay *in vitro.* The recombinant KLF5-6×His protein was expressed and purified from *E.coli* and GST-ATXN3L was expressed and purified from HEK293FT cells. GST was used as a negative control. **F.** GST-ATXN3L directly interacted with KLF5-6×His as measured by the GST pull-down assay *in vitro.* Both recombinant KLF5-6×His and GST-ATXN3L proteins were expressed and purified from *E.coli*.

To examine whether ATXN3L physically interacts with KLF5, we transfected Myc-ATXN3L and KLF5-3×Flag into HEK293FT cells and immunoprecipitated KLF5-3×Flag. As shown in Figure 2A, Myc-ATXN3L was co-immunoprecipitated with KLF5-3×Flag. Additionally, we detected the protein interaction between Myc-ATXN3L and endogenous KLF5 in SUM1315 cells by immunoprecipitation (Figure 2B).

Next, we mapped which domain of KLF5 is responsible for the interaction by generating 3 truncated KLF5 fragments (Figure 2C). Full-length KLF5 (1-457), the N-terminus of KLF5 (1-200) and the C-terminus of KLF5 (367-457) were shown to interact with Myc-ATXN3L, whereas the middle part of KLF5 (201-366) did not. These results suggest that both ends of KLF5 participate in the protein interaction with ATXN3L. Furthermore, we tried to identify which region of ATXN3L is responsible for the interaction by generating a series of 4 GST-fused ATXN3L deletion mutants. By GST pull-down assays, full-length ATXN3L (1-380), the N-terminus of ATXN3L (1-212) and the C-terminus of ATXN3L (285-380) interacted with KLF5-3×Flag (Figure 2D). The middle part of ATXN3L (213-284) did not interact with KLF5-3×Flag (Figure 2D). These results suggest that both ends of ATXN3L participate in the protein interaction with KLF5.

Finally, to test whether ATXN3L directly interacts with KLF5, we expressed and purified recombinant proteins, including KLF5-6×His from *E.coli* and GST-ATXN3L from both HEK293FT cells and *E.coli*. By GST pull-down assays, we showed that the purified GST-ATXN3L protein pulled down the purified KLF5-6×His protein *in vitro* (Figure 2E-2F).

### ATXN3L stabilizes KLF5

**Figure 3:**
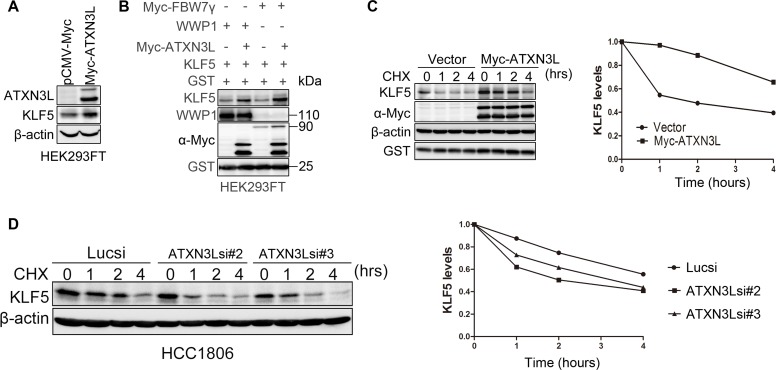
ATXN3L stabilizes KLF5 **A.** ATXN3L increased the KLF5 protein level in HEK293FT cells. Myc-ATXN3L and pCMV-Myc empty vector were transfected together with KLF5 into HEK293FT cells. The KLF5 protein level was increased by Myc-ATXN3L. **B.** ATXN3L increased the KLF5 protein level in the presence of WWP1 and FBW7 in HEK293FT cells. HEK293FT cells were transfected with KLF5, E3 ligases (WWP1, Myc-FBW7γ or an empty vector) and Myc-ATXN3L (or the empty vector). GST was used as the transfection negative control. **C.** ATXN3L increased the KLF5 protein stability. KLF5 and Myc-ATXN3L were co-expressed in HEK293FT cells. After the cells were treated with CHX for different time periods, KLF5 protein levels were analyzed by WB. Quantitative results are shown on the right. The KLF5 protein half-life was extended by at least two hours. **D.** ATXN3L knockdown decreased the KLF5 protein stability in HCC1806. Quantitative results are shown on the right.

As described above, knockdown of ATXN3L decreased the endogenous KLF5 protein levels in both HCC1806 and SUM1315 cell lines (Figure 1C & 1E). When Myc-ATXN3L was over-expressed in HEK293FT, the KLF5 protein level was elevated (Figure 3A). We previously demonstrated that the E3 ubiquitin ligases, such as WWP1 and SCF^Fbw7^, target the KLF5 protein for ubiquitin-mediated degradation [10, 11]. To test whether ATXN3L can antagonize E3 ligase-mediated KLF5 degradation, we transfected WWP1, Myc-FBW7, KLF5 and ATXN3L into HEK293FT cells. As expected, ATXN3L over-expression blocked the KLF5 protein degradation induced by WWP1 and FBW7 (Figure 3B).

To further determine whether ATXN3L increases the KLF5 protein stability, we measured the KLF5 protein half-lives by the cycloheximide (CHX) chase assays [9]. Notably, ATXN3L overexpression dramatically extended the KLF5 protein half-life (Figure 3C-3D). Furthermore, we knocked down ATXN3L by two different siRNAs in HCC1806 and measured the KLF5 protein half-lives by CHX chase assays. As expected, knockdown of ATXN3L obviously shortened the KLF5 protein half-life (Figure 3D). Collectively, these results suggest that ATXN3L stabilizes the KLF5 protein.

### ATXN3L deubiquitinates KLF5

**Figure 4:**
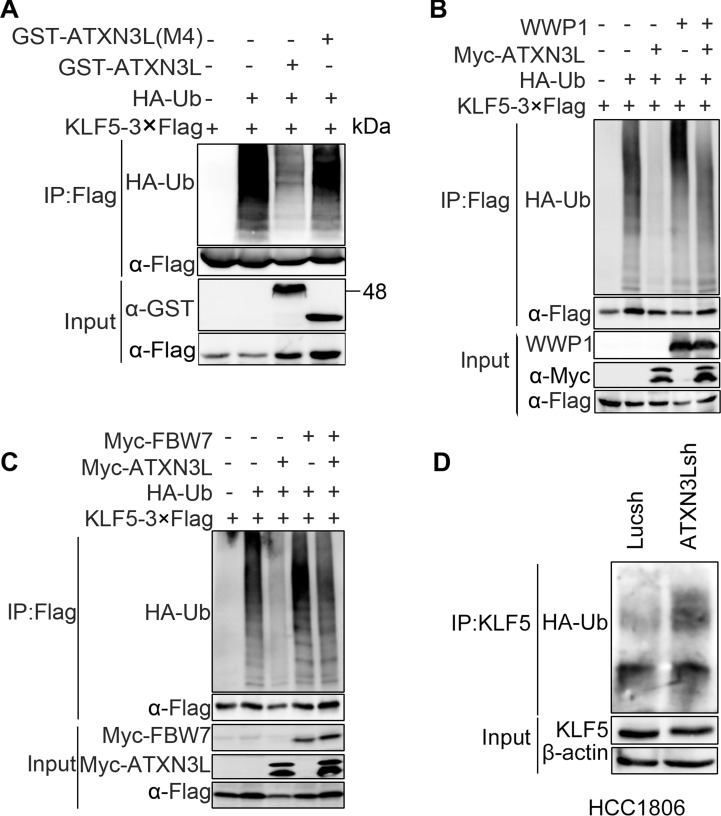
ATXN3L deubiquitinates KLF5 **A.** ATXN3L decreased the polyubiquitination of KLF5-3×Flag in a DUB activity dependent manner. KLF5-3×Flag and HA-Ub were co-expressed with GST-ATXN3L or GST-ATXN3L (M4, without the catalytic josephin domain) in HEK293FT. The cells were treated with MG132 for 6 h. **B.** ATXN3L antagonized the WWP1-mediated KLF5 polyubiquitination in HEK293FT cells. WWP1 increased the polyubiquitination of KLF5. **C.** ATXN3L antagonized the FBW7-mediated KLF5 polyubiquitination in HEK293FT cells. Myc-Fbw7γ increased the polyubiquitination of KLF5. **D.** ATXN3L knockdown increased the endogenous KLF5 protein ubiquitination in HCC1806. ATXN3L was stably knocked down by ATXN3Lsh#2. HA-Ub was overexpressed to facilitate the detection of the endogenous KLF5 protein ubiquitination.

We then sought to determine whether ATXN3L directly decreases the KLF5 ubiquitination. We transfected KLF5-3×Flag, HA-Ub, and GST-ATXN3L or GST-ATXN3L (M4, deletion of the Josephin DUB catalytic domain) into HEK293FT cells and treated the cells with the proteasome inhibitor MG132 to block protein degradation. Polyubiquitinated KLF5 proteins were detected using an anti-HA antibody after immunoprecipitation using the anti-Flag antibody. GST-ATXN3L, but not GST-ATXN3L(M4), dramatically decreased the KLF5 protein polyubiquitination (Figure 4A).

Moreover, we tested whether ATXN3L antagonizes the E3 ubiquitin ligase mediated KLF5 ubiquitination. We transfected KLF5-3×Flag, HA-Ub, Myc-ATXN3L and WWP1 or Myc-FBW7 into HEK293FT cells and treated the cells with MG132. As expected, ATXN3L decreased both E3 ligases mediated KLF5 ubiquitination (Figure 4B-4C).

Finally, we measured the endogenous KLF5 ubiquitination after ATXN3L was knocked down in HCC1806. As shown in Figure 4D, ATXN3L silencing increased the endogenous KLF5 protein ubiquitination. Collectively, these results suggest that ATXN3L is a specific DUB for KLF5 and that ATXN3L increases KLF5 protein stability through deubiquitination.

### ATXN3L promotes breast cancer cell proliferation partially through KLF5

**Figure 5:**
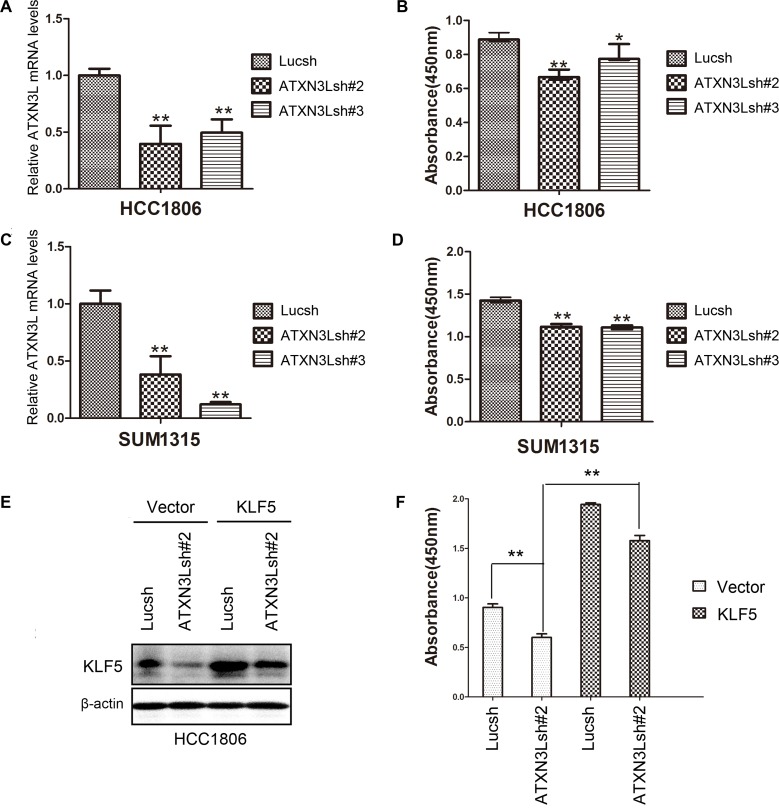
Knockdown of ATXN3L significantly suppresses breast cancer cell proliferation **A.** The knockdown effect of ATXN3L using two independent shRNAs in HCC1806 cells was determined by RT-qPCR at the mRNA level. **, *p* < 0.01, *t*-test. **B.** Stable knockdown of ATXN3L decreased the proliferation of HCC1806 cells, as measured by CCK8. *, *p* < 0.05, **, *p* < 0.01, t-test. **C.** The knockdown effect of ATXN3L using two independent shRNAs in SUM1315 cells was determined by RT-qPCR at the mRNA level. **D.** Stable knockdown of ATXN3L decreased the proliferation of SUM1315 cells, as measured by CCK8. **E.** KLF5 was stably overexpressed in ATXN3L knockdown HCC1806 and control cells, as detected by WB. **F.** KLF5 overexpression rescued the ATXN3L knockdown induced growth arrest in HCC1806, as measured by CCK8.

Our previous studies demonstrated that KLF5 promoted breast cancer cell proliferation [1]. However, the function of ATXN3L in breast cancer is unknown. Since ATXN3L stabilizes KLF5 and inhibits the expression of p27 and p21, it is plausible that ATXN3L also promotes breast cancer cell proliferation. We generated ATXN3L stable knockdown HCC1806 and SUM1315 cell lines. RT-qPCR confirmed that both ATXN3L shRNAs significantly reduced the *ATXN3L* mRNA levels in HCC1806 and SUM1315 cell lines (Figure 5A & 5C). Consistently, knockdown of ATXN3L by shRNAs significantly decreased cell proliferation in both breast cancer cell lines, as measured by CCK-8 (Figure 5B & 5D). Interestingly, knockdown of ATXN3L in HCC1806 did not affect cell migration, as measured by wound healing and transwell assays (Figure S1)

To test whether ATXN3L functions through KLF5, we stably overexpressed KLF5 in ATXN3L knockdown HCC1806 cells. As expected, KLF5 overexpression significantly promoted cell proliferation and rescued the ATXN3L knockdown induced cell growth inhibition (Figure 5E-5F). These results indicate that endogenous ATXN3L promotes breast cancer cell proliferation partially through stabilizing KLF5.

## DISCUSSION

Our previous studies suggest that KLF5 promotes breast cancer cell proliferation by regulating a number of target genes, such as *p21*, *p27*, *Cyclin D1*, *FGF-BP* and *mPGES1* [18]. It is important to understand the upstream regulatory mechanism for KLF5. At the transcriptional level, we previously reported that KLF5 is induced by progesterone via the progesterone receptor [6]. At the post-translational level, the KLF5 protein is degraded by mechanisms involving WWP1 and SCF^FBW7^ E3 ligases [10, 11]. In addition, the KLF5 protein degradation is inhibited by YAP and TAZ [19, 20] but increased by WWOX [21]. In this study, we identified ATXN3L as a KLF5 DUB and thus a stabilizer of KLF5.

We showed that ATXN3L depletion decreased and over-expression increased the KLF5 protein levels. The ATXN3L protein interacted with the KLF5 protein and decreased the KLF5 polyubiquitination. Functionally, depletion of ATXN3L increased the p27 and p21 protein levels and suppressed breast cancer cell proliferation. Thus, ATXN3L is a novel positive regulator of KLF5 and may serve as a novel therapeutic target.

The function of ATXN3L has never been well characterized. ATXN3L belongs to the the Josephin DUB sub-family, which contains four members, such as ATXN3L, ATXN3, JOSD1 and JOSD2. The Josephin domain is a conserved cysteine protease domain. ATXN3 and ATXN3L share the 85% sequence identity in the Josephin domains. Interestingly, ATXN3L is the most active DUB enzyme among the four members [22]. ATXN3 has been shown to restrict PTEN transcription in lung cancer cells [23]. Additionally, ATXN3 is a potential therapeutic target in neurodegenerative disease [24]. JOSD1 was recently shown to regulate membrane dynamics, cell motility and endocytosis [25]. ATXN3L has been reported to promote migration of A549 cells [26]. Our findings in this study suggest that ATXN3L promotes cell proliferation but not cell migration. Although ATXN3L decreases the KLF5 protein ubiquitination, ATXN3L may have other substrate proteins. The physiological and pathological roles of ATXN3L require further investigation in the future.

In this study, we failed to detect the ATXN3L protein expression because of the lacking of a functional antibody. Using RT-qPCR, we measured the *ATXN3L* mRNA levels in a panel of breast cancer cell lines and tumors but failed to observe any significant expression changes between normal and cancer samples (Figure S2A-B). Based on the TCGA database, there is no expression correlation between *ATXN3L* and *KLF5* at the mRNA level (Figure S2C). To our surprise, a high level of *ATXN3L* mRNA is significantly correlated with a long relapse free survival in basal breast cancer patients (Figure S2D). Since ATXN3L regulates KLF5 at the protein level, it will be significant to develop a good anti-ATXN3L Ab to examine the ATXN3L protein expression in breast tumors in order to assess the clinical relevance of ATXN3L.

The KLF5 protein is ubiquitinated by multiple E3 ubiquitin ligases, such as WWP1, SCF^FBW7^, Smurf2, and so on. Similarly, KLF5 is likely to be stabilized by other DUBs besides ATXN3L. Whether the deubiquitination of KLF5 protein by ATXN3L is regulated is unknown at present. Nevertheless, ATXN3L increased the KLF5 protein stability and inhibited the expression of KLF5 downstream target genes, such as p21 and p27, and promote basal type breast cancer cell proliferation. It is possible that ATXN3L serves as a novel therapeutic target for breast cancer and other cancer patients.

In summary, our study identified ATXN3L as a KLF5 DUB in breast cancer and found that ATXN3L stabilized the KLF5 protein and promoted cell proliferation partially through KLF5. Our findings uncovered a new regulatory mechanism for KLF5 and suggest that ATXN3L is a potential therapeutic target for breast cancer treatment.

## MATERIALS AND METHODS

### DUB siRNA library screening

The siRNA library consisting of 87 human DUBs (#4392425) was purchased from Applied Biosystems (Ambion Silencer siRNA Custom Library). Different siRNAs were transfected with lipofectamine 2000 (Invitrogen, Carlsbad, CA) into HeLa cells in 24-well plates for two days. Cell lysates were extracted and the protein levels of endogenous KLF5 were measured by Western blotting (WB).

### Antibodies

The anti-ATXN3L (GTX51610) rabbit polyclonal antibody was purchased from GeneTex Inc. (San Antonio, TX). The anti-KLF5 rabbit polyclonal antibody has been described previously [23], and the anti-p27^Kip1^ (#610241) mouse antibody was purchased from BD Transduction Laboratories (San Diego, CA). The anti-p21^Cip1/Waf1^ (#2947) rabbit polyclonal antibody was purchased from Cell Signaling Technology (Danvers, MA). The anti-HA (Y-11, sc-805) rabbit polyclonal antibody and the anti-Myc (9E10, sc-40) mouse monoclonal antibody were obtained from Santa Cruz Biotechnology (Santa Cruz, CA). The anti-GST (G7781) rabbit monoclonal, anti-Flag (F7425) and anti-β-actin (AC-15, A5441) mouse monoclonal antibodies were purchased from Sigma-Aldrich (St. Louis, MO). The anti-WWP1 (H00011059-M01) rabbit polyclonal antibody was purchased from Abnova (Taiwan).

### Cell culture and plasmid transfection

HCC1806 breast cancer cells were cultured in RPMI-1640 (with 2 mM L-glutamine) supplemented with 5% fetal bovine serum (FBS), and 1% penicillin/streptomycin (P/S). SUM1315 breast cancer cells were cultured in F-12 medium supplemented with 5% FBS, insulin (5 μg/ml), epidermal growth factor (10 ng/ml) and 1% P/S. Human embryonic kidney HEK293FT cells and HeLa cervical cancer cells were maintained in Dulbecco's Modified Eagle's Medium (DMEM) containing 5% FBS and 1% P/S.

All plasmids were transfected into HEK293FT cells using lipofectamine 2000 following the manufacturer's instructions. The siRNA and shRNA target sequences used in this study are shown in Supplementary Table 1. pSIH-H1-Lucsh or pSIH-H1-ATXN3Lsh and packaging plasmids were transfected into HEK293FT cells using lipofectamine 2000. Lentiviruses were collected 48-72 h after transfection and used to transduce HCC1806 and SUM1315 cells. The puromycin resistant cell populations were selected 48 h after transduction.

### Protein purification

ATXN3L was subcloned into the prokaryotic GST-fused expression vector pGEX-6p-1. The constructs were transformed into *E. coli* BL21 (DE3) and 1 mM IPTG was added to induce the expression of the recombinant protein at 16°C. The cells were harvested, resuspended in 50 ml lysis buffer containing 100 mM Tris-HCl, pH 8.0,100 mM NaCl, 50 mM EDTA, 2 % TritonX-100, 1.2 μg/ml PMSF, 5 mM DTT, and 1.67 mg/ml lysozyme for 30 min and sonicated. The GST fusion proteins were affinity purified with glutathione sepharose 4B and eluted with elution buffer (100 mM Tris-HCl, pH 8.0 and 10 mM glutathione). The purified protein was analyzed by 10% SDS-PAGE with Coomassie blue staining.

KLF5 was subcloned into the pET28-3C vector. The plasmid was transfected into BL21(DE3) cells to express the protein as described above. The cells were harvested in lysis buffer (20 mM Tris, pH 8.0, 0.2 mM β-mercaptoethanol, and 500 mM NaCl) and purified with a Ni-NTA (Qiagen) column.

### Protein-protein interaction assays

Immunoprecipitation using anti-Flag M2 agarose beads (A2220, Sigma) and GST pull-down using the Glutathione-Sepharose 4B slurry beads have been described previously [10]. Briefly,

HEK293FT cells were transfected with expression plasmids in 6-well plates for 48 h. The cells were collected into 0.25 ml of 1×ice-cold cell lysis buffer (50 mM Tris-Cl, pH 7.4, 150 mM NaCl, 1 mM EDTA, 1% Triton X-100, 1% protease inhibitor cocktail (P8340, Sigma)) and incubated on ice for 30 min. Then, cell lysates were centrifuged at 10, 000 g for 15 min at 4°C. The supernatant (200 μl) were incubated with 30 μl of anti-Flag M2 agarose beads or anti-Myc antibody plus protein A/G agarose (for endogenous KLF5 protein interaction) overnight at 4°C with gentle rocking. For the GST pull-down assay, the Glutathione-Sepharose 4B slurry beads were incubated with the GST-ATXN3L cell lysate or purified GST-ATXN3L protein and the purified KLF5-6×His protein overnight at 4°C. The beads were washed four times with 1 ml of 1 × cell lysis buffer or pre-cooled PBS with 1% TritonX-100. Proteins were subjected to WB.

### Deubiquitination assays

HEK293FT cells were transiently transfected with HA-Ub and other plasmids in 6-well plates. Two days after transfection, the cells were harvested in 150 μl SDS lysis buffer (50 mM Tris-Cl, pH 6.8, 1.5% SDS). The samples were boiled for 15 min. 100 μl of protein lysate was diluted with 1.2 ml EBC/BSA buffer (50 mM Tris-Cl, pH 6.8, 180 mM NaCl, 0.5% CA630, 0.5% BSA) and incubated with 30 μl anti-Flag M2 agarose beads or anti-KLF5 antibody plus protein A/G agarose (for endogenous KLF5 protein ubiquitination) overnight at 4°C with rotation. The beads were collected by centrifugation at 10, 000 g for 1 min at 4°C and washed three times with 1 ml ice-cold EBC/BSA buffer. Proteins were resuspended with 15 μl of 3 ×SDS sample buffer and analyzed by WB.

### RT-qPCR assays

Total RNAs were isolated using RNeasy Mini Kit (Qiagen, Hilden). Reverse transcriptions were performed using the Iscript cDNA synthesis kit (Bio-Rad, Hercules, CA). Quantitative RT-PCR was performed on an ABI-7900 system using SYBR Green reagents (ABI, Austin, TX). Primers used for ATXN3L and GAPDH are shown in Supplementary Table 2.

### Cell-proliferation assays

Cell-proliferation assays were performed using Cell-Counting Kit-8 (CCK-8, Dojindo, Kumamoto, Japan). ATXN3L stable knockdown SUM1315 and HCC1806 and control cells were seeded in 24-well plates and maintained at 37°C in a humidified incubator. After two days, the culture media were replaced with 500 μl fresh media with 50 μl CCK-8 solution. The cells were incubated for 1.5 h and absorbance was measured at 450 nm.

### Cell migration assays

HCC1806 cells were plated in 12-well plates for wound-healing assays. For the Transwell assays (Costar, #3422), HCC1806 cells were placed on the upper layer of a cell permeable membrane and media containing 10% FBS was placed in the lower chamber. Following an incubation period, the cells that had migrated through the membrane were stained and counted.

### Statistical analysis

All analyses were performed using the SPSS 13.0 statistical software package. Both the cell-proliferation assays and quantitative RT-PCR assays were conducted in quadruplicate.

When appropriate, the resulting data were pooled and expressed as mean±standard deviation and analyzed by t-test. P values less than 0.05 were considered to significant.

## SUPPLEMENTARY MATERIAL FIGURES AND TABLES


