# Supplementary material for: Ataxin-3 like (ATXN3L), a member of the Josephin family of deubiquitinating enzymes, promotes breast cancer proliferation by deubiquitinating Krüppel-like factor 5 (KLF5)
Source: Oncotarget. 2015 May 12;6(25):21369–78. doi: 10.18632/oncotarget.4128 (PMC4673271; doi:10.18632/oncotarget.4128)
Supplement: Supplementary file 1 [file oncotarget-06-21369-s001.docx]

Ataxin-3 like (ATXN3L), a member of the Josephin family of deubiquitinating enzymes, promotes breast cancer proliferation by deubiquitinating Krüppel-like factor 5 (KLF5)

**Supplementary Material**

**Figure S1: ATXN3L does not regulate cell migration in**

**HCC1806**

**A.** ATXN3L stable knockdown does not affect wound healing.

ATXN3L was stably knocked down in HCC1806 cells. The cells were plated in 12-well plates for 24 h wound-healing assays. Quantitative results are shown on the right.

**B.** ATXN3L stable knockdown does not affect transwell migration.

The cells were placed on the upper layer of a cell permeable membrane and media containing 10% FBS was placed in the lower chamber. Following an incubation period (48-72 h), the cells that had migrated through the membrane were stained and measured. Quantitative results are shown on the right.

**Figure S2: The mRNA expression of *ATXN3L* in breast cell lines and tumors.**

**A.** The mRNA expression levels of *ATXN3L* in 9 breast cancer cell lines and the MCF10A immortalized breast epithelial cell line, as measured by RT-qPCR.

**B.** The mRNA expression levels of *ATXN3L* in 21 breast tumors (13 Luminal, 3 HER2+ and 5 TNBC) and two normal breast tissues, as measured by RT-qPCR.

**C.** The mRNA expression levels of *ATXN3L* and KLF5 in breast tumors in the TCGA database are not correlated.

**D.** A high level of *ATXN3L* mRNA is significantly (logrank p=0.004) associated with a long relapse free survival in basal breast tumors. Kaplan-Meier plotter was used to analyze the breast cancer RNA seq data from the TCGA database.
